# Occupational therapy discharge planning for older adults: A protocol for a randomised trial and economic evaluation

**DOI:** 10.1186/1471-2318-12-34

**Published:** 2012-07-08

**Authors:** Kylie Wales, Lindy Clemson, Natasha A Lannin, Ian D Cameron, Glenn Salked, Laura Gitlin, Laurance Rubenstein, Sarah Barras, Lynette Mackenzie, Collette Davies

**Affiliations:** 1 Ageing Work and Health Research Unit, Faculty of Health Sciences, The University of Sydney, PO BOX 170, Lidcombe, NSW, 2141, Australia; 2 Occupational Therapy Department, Alfred Health, Melbourne, Victoria Australia; Faculty of Health Sciences, Alfred Clinical School, La Trobe University, The Alfred 55 Commercial Road, Prahan, VIC, Australia; 3 Rehabilitation Studies Unit, Sydney Medical School, The University of Sydney, PO BOX 6, Sydney, NSW, 2112, Australia; 4 School of Public Health, The University of Sydney, Edward Ford Building, Sydney, NSW, 2006, Australia; 5 Johns Hopkins University, 525 Wolf Street, Baltimore, MD, 21205, USA; 6 The Donald W. Reynolds Chair in Geriatric Medicine, The University of Oklahoma, HSC, 1122 NE 13th Street, ORB 1200, Oklahoma, OK, USA; 7 Ramsay Healthcare. Group Clinical Governance Unit. Casemix Consultant, 11 Standard Avenue, Box Hill, 3128, VIC, Australia

**Keywords:** Activities of daily living, Home assessment, Ageing, Randomised trial, Discharge planning, Occupational therapy

## Abstract

**Background:**

Decreased functional ability is common in older adults after hospitalisation. Lower levels of functional ability increase the risk of hospital readmission and nursing care facility admission. Discharge planning across the hospital and community interface is suggested to increase functional ability and decrease hospital length of stay and hospital readmission. However evidence is limited and the benefits of occupational therapists providing this service has not been investigated.

This randomised trial will investigate the clinical effectiveness of a discharge planning program in reducing functional difficulties of older adults post-discharge. This trial will also examine the cost of the intervention and cost effectiveness when compared to in-hospital discharge planning.

**Methods/design:**

400 participants admitted to participating hospitals will be recruited. Participants will be 70 years of age and over, have no significant cognitive impairment and be independently mobile at discharge. This study protocol was approved by the ethics committee of Ryde Rehabilitation Human Research Ethics Committee, Western Sydney Local Health District (Westmead Campus) Human Research Ethics Committee, Alfred Health Human Research ethics committee for the randomised trial and NSW Population and Health Service Human Research Ethics Committee for data linkage. Participants will provide informed written consent.

Participants will be randomly allocated to the intervention or control group. The intervention group will receive discharge planning therapies primarily within their home environment while the control group will receive an in-hospital consultation, both provided by trained occupational therapists. Primary outcome measures will be the Nottingham Extended Activities of Daily Living Scale (NEADL) and the Late Life Disability Index (LLDI) which will measure functional independence, and participation and limitation in daily life activities.

**Discussion:**

This trial will investigate the effectiveness and cost effectiveness of occupational therapy discharge planning in reducing functional difficulties. Results will have a direct impact on healthcare practice and policy.

**Trial registration:**

ACTRN12611000615987.

## Background

An ageing population with an increasing life expectancy has placed significant demand on the healthcare system across the developed world [1,2]. Older adults are the main users of the acute healthcare system in Australia, with higher numbers of hospital admissions than any other age group [3]. Rising healthcare costs, increased consumer choice and a focus on aging in place have further increased the demand on the healthcare system [4]. Not surprisingly, older adults are the target of numerous health care policies as a means to reduce this impact on the healthcare system and to enhance outcomes for older adults [4,5].

During hospital admissions, older adults are at significant risk of functional decline and are often discharged at a lower level of functional ability than they had upon admission [6]. Functional decline may lead to an increased need for services, lower levels of autonomy, readmission to hospital, or nursing care facility admission [7]. Discharge planning is considered the best way to support the older adult to return home to pre-hospital function [8,9]. The purpose of discharge planning is to enable both the health professional and older adult to work together to plan their return home, identify any needs and organise support for after discharge [9,10]. Successful discharge planning may also reduce hospital length of stay, readmission rates and, caregiver burden and enhance the coordination of services [8-10]. Results from two systematic reviews identify that discharge planning across the hospital and community environments show positive effects [8,9]. However these results are based on lesser quality studies and conclude that further large randomised trials are required to comprehensively establish this outcome [8,9].

Occupational therapists are involved in discharge planning as they consider older adults’ abilities to independently and safely function within their own environment [11]. Assessments of the older adult’s functional ability may occur either within the hospital setting and/or within the home environment, generally during a pre-discharge home assessment [12]. Yet, a lack of research has resulted in varying practices by occupational therapists for discharge planning.

In-hospital consultations are commonly provided in countries such as America, where subsidised healthcare does not support occupational therapy home visiting [12]. In-hospital consultations enable the therapist to make decisions regarding an older persons ability to carry out ADL and IADL’s within their home environment and decisions on assistive technology [12]. However, in-hospital consultations can be problematic in obtaining a true reflection of an older person’s home environment and their ability to function within this environment.

Research indicates that home assessments enable a contextually relevant reflection of the older adult’s functional ability [13,14]. In countries such as Australia and the United Kingdom, pre-discharge home assessments are part of standard practice for occupational therapists working in aged care [15-17]. Despite being commonly provided, a survey of occupational therapy departments in Australia highlighted that assessments are not conducted consistently across departments [15]. The decision to carry out a home assessment can be policy driven, influenced by staffing levels and reliant on the clinical background and expertise of the individual occupational therapist [11,15]. As a result, inconsistencies in home assessment procedures exist and non-standardised evaluation of outcomes is typical [11,12]. Results of research also suggested that post-discharge home assessments may enhance the discharge planning process [14]. Whether or not hospital consultations and/or home assessments are included in part of discharge planning practice is dependent on results from high quality research.

Results of two feasibility trials [16,18] indicate that a large randomised trial to determine the effectiveness of occupational therapy discharge planning is achievable. These and other studies [15,19] support the necessity of evaluating cost effectiveness of occupational therapy discharge planning in addition to clinical effectiveness of home assessments in reducing functional difficulties. Through cost effectiveness analysis, decisions can be made as to whether a new intervention is effective and whether or not benefits are cost efficient compared to current practice [20]. Since occupational therapy treatment focuses on increasing or maintaining independence, there is a potential for discharge planning to reduce healthcare costs, including readmission to hospital, decreased community health service use and delay nursing care facility admission.

Research must be conducted to identify the best practice for occupational therapy discharge planning and this should be evaluated for its effectiveness and cost effectiveness. The HOME intervention has been developed from recommendations in occupational therapy literature as a method of comprehensive discharge planning and now requires evaluation. The primary aim of this study is to determine the effectiveness of occupational therapy discharge planning in reducing functional difficulties. The primary hypotheses is that people who receive the HOME intervention will have (i) higher levels of functional independence three months after discharge then the control group and (ii) higher levels of participation and resumption of usual life activities compared to the control group.

## Methods/design

A multicentre trial of n = 400 participants admitted to four participating hospitals in Australia will be conducted. Screening will be conducted by a research assistant. Participants will be eligible for inclusion if they: 1) are 70 years or older, 2) are expected to return to a community dwelling after discharge, 3) have no significant cognitive impairment (score < 5 errors on Short Portable Mental Status Questionnaire [21], and 4) are conversant in English. Participants will be excluded if they: 1) score < 5 on locomotion sub score of the Functional Independence Measure^TM^[22], 2) are expected to require a wheelchair at discharge, 3) have received a comprehensive occupational therapy home assessment within the last 6 months, or 4) have significant co-morbidities (Score ≥8 age adjusted, on the Charlson Co-morbidity index [23]).

### Randomisation

**Figure 1:**
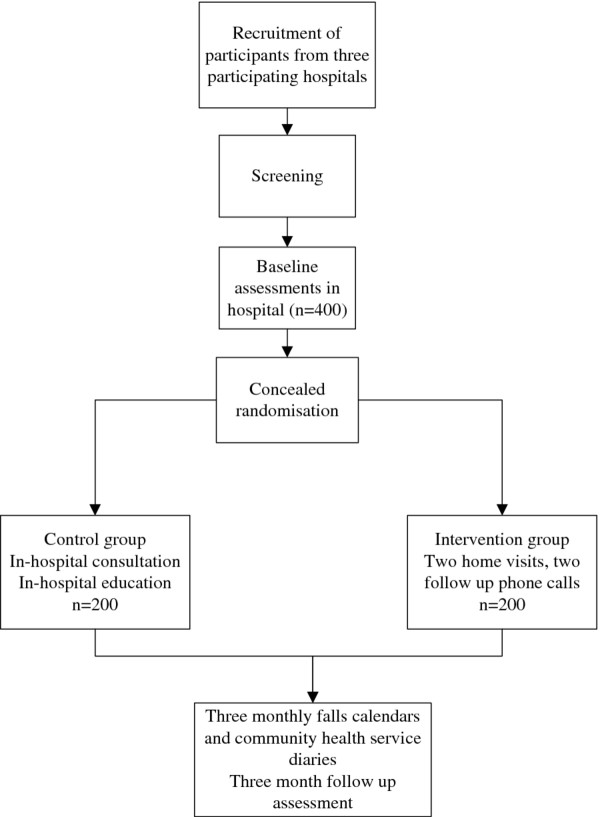
Flow of participants through the HOME trial.

Individual randomisation will occur following baseline assessment, as demonstrated in Figure 1. Participants will be stratified by site and by age (70–84, >84 years). Age stratification has been incorporated as the research group expects that the HOME intervention will have differential effects on these age groups. The randomisation schedule was developed by a researcher not involved in group allocation and using the program STATA ver. 17 and the add-on program Ralloc [24] to generate the random sequence. Once baseline assessments have been completed allocation will be determined by using a password protected website to conceal randomisation. This will be carried out by a researcher not involved in follow up assessment. Occupational therapists administering the experimental and control interventions will not be blind to allocation.

### Intervention

Participants randomised to the intervention group will receive the HOME protocol based on recommendations of occupational therapy literature. The aims of the HOME protocol are outlined in Table 1. The HOME protocol is primarily conducted in the participant’s home environment and is carried out by the same occupational therapist to promote continuity of care. There is a focus on functional ability, safety and transition from hospital to home throughout the HOME intervention. All occupational therapists implementing the HOME intervention will undergo training in the HOME protocol. Training will include two sessions relating to assessments of the persons functional ability, goal setting, and home assessment. The Westmead Home Safety Assessment [25] will be included as part of training to increase occupational therapists awareness of potential environmental barriers and hazards. On-going monthly training will be provided throughout the program as identified by project manager and therapists. Fidelity checks will be incorporated throughout the study to ensure protocol adherence by occupational therapists and to monitor program quality. The fidelity checks will be conducted by independent expert occupational therapists trained in the HOME protocol. The independent therapists will attend a home visit with a participant and treating occupational therapist. They will use a checklist to determine adherence to the protocol by the treating occupational therapist.

**Table 1:** Aims from the HOME occupational therapy discharge planning protocol

	
**1.Prepare the person to return home and resume their desired lifestyle.**	
a. Assess the individual person’s occupational needs respecting their personal beliefs, needs and goals and understand the older person’s patterns of daily living [26]	
b. Recommend functional adaptations that will maximise the person’s abilities as they reintegrate back to usual living [27]. The aim is for pre-admission standard of living [28]	
c. Optimise the person-environment fit [29]	
d. Recommend and implement environmental modifications	
e. Prescribe adaptive equipment and observe its use insitu [26]	
**2.Enhance self-efficacy beliefs and promote independence and sense of control through mastery of meaningful tasks**	
1. Transfer altered skills to the home situation and assist in the adjustment to these changes [30]	
2. Habitual retraining insitu using strategies such as situational cues and targeting behaviours for change	
3. Encourage one-on-one education about the safe performance of activities in and around their home and immediate community	
4. Facilitate joint problem solving and solution generation [26,30]	
5. Lessen a person’s fear during the transition from hospital to home [11]	
**3.Use goal setting as a therapeutic tool**	
1. Develop client centred goals [31] that address individual occupational needs [26]	
2. Develop goals that aim to maximise the person’s potential to participate in desired activities [27]	
3. Include goals which enable the person to participate in activities both in the home and in the community [27] and incorporate health and physical activity goals [32]	
4. Plan for increasing independence/capacity over the next three months, setting goals for increasing ability [12,14,30]	
5. Review progress towards goals and facilitate further joint problem solving	

In hospital, the occupational therapist will focus on rapport building with the participant and family members. Information will be gathered about the participant’s home environment and functional ability in preparation for the pre-discharge home assessment. Collaborative goal setting [12,31] and joint problem solving [26] will be used as a means to plan safe discharge. Through understanding the participant’s experience and situation the therapist has an understanding of how this may influence goals and therefore the overall occupational therapy intervention [33]. The goals and methods of occupation-based therapy spring from a collaboration between the general match between environments and persons with various patterns of abilities and disabilities, and with consideration for personal ownership of goals and methods [34].

In the next phase, the occupational therapist will conduct a pre-discharge home assessment with the participant approximately five days prior to expected discharge or at a time the participant has returned to sufficient functioning for a home assessment [35]. During the pre-discharge home assessment the therapist will jointly evaluate the environment, issues and problems with the participant and family.

A post-discharge home assessment will be conducted within the first week following discharge. This visit will be based on in-home training and to follow up on any unmet needs of the participant. The therapist will shift focus to long term independence by consolidating transitional goals with goals that focus on enhancing functional independence and resumption of meaningful activities. The therapist should not be restricted to planning interventions based on reason for admission, rather taking into account the broader person environment fit. Two follow up telephone calls will be provided at two and four weeks post-discharge to provide ongoing support to participant and family. The participant will be encouraged to independently problem solve in preparation for the close of the therapeutic relationship.

### Control group

Participants randomised into the control group will receive a hospital-based discharge planning assessment by a hospital occupational therapist. During this assessment information regarding the participant’s ability to carry activities of daily living, instrumental activities of daily living and their home environment will be gathered. This information will be used to plan for discharge, including assistive equipment and home modification needs. Participants in the control group will not receive an occupational therapy home assessment. If the occupational therapist identifies a need for home modifications, the research assistant, also a qualified occupational therapist, will assess the participant’s home for suitability of recommended modifications and provide this feedback to the treating therapist. The participant will not attend this home assessment to reduce the possibility of contamination.

All other medical and allied health treatments will be conducted as per usual practice for both groups.

### Outcome measurement

Data will be directly collected from medical records, participant self-report, calendars, standardised functional assessments and data linkage.

Participant self-report and medical records will be used to identify suitability for inclusion. Baseline assessments will then be completed by a research assistant with the participant in hospital. This assessment will involve functional questionnaires, demographic information, health related quality of life and falls history/self efficacy. Each baseline assessment will take approximately one hour to complete. These assessments will be completed again at three months after discharge from hospital by a blinded research assistant with the participant in their own home.

At discharge, all participants will receive a set of monthly calendars and a community health service use diary. Participants will be asked to record daily if they have a fall or not or if they use any community health services for three months after hospitalisation. Participants will be telephoned fortnightly post-discharge to follow up on any falls, community health service use, nursing care facility admission, and any out of pocket expenses to enhance accuracy of data. Participants will be prompted to return these to research staff during their final assessment. The assessments and telephone calls will be completed by a research assistant blinded to group allocation.

Primary outcome measures: Functional independence will be assessed using the Nottingham Extended Activities Daily Living scale (NEADL) [36] while participation and limitation in activities of daily living will be assessed using the Late Life Disability Index (LLDI) [37]. These assessments will be completed at baseline and three months post-discharge from hospital.

Secondary outcome measures: Fear of falling during activities of daily living will be measured using the International Falls Efficacy Scale (FES-I) [38]. Physical activity will be measured using the question “do you get out of the house as often as you like?” (used previously in a trial) [39] and the physical activity sub-scale of the SF-36v2 [40]. Health related quality of life will be assessed using the SF-12v2 [40]. Falls and community health service use will be recorded using a monthly self report falls calendar and diary.

Cost effectiveness measures: Functional independence will be valued using the NEADL scale [36], whereas health outcomes will be valued as quality adjusted life years (QALYs) [20]. QALYs will be derived from the SF-12v2 converted to SF-6D [20]. Changes in the NEADL scale will be expressed as the proportion of trial respondents who achieve a clinically significant improvement on the NEADL scale.

The main costs of treatment will be determined using a micro-costing approach and will include occupational therapist time spent, equipment and home modifications prescribed, community health service use, nursing care facility admission, general practitioner visits and hospital readmissions and length of stay [41]. The occupational therapist will keep a log of all time spent administering the HOME intervention, and details of recommendations. Participants will be asked to document community and health service use in a diary. A cost effectiveness questionnaire will be completed at their three month follow up to investigate community health service use further. Data linkage will be used to follow up any hospital admissions, length of stay and death post-discharge. Hospital bed costs will be calculated using case mixed costs defined from the Australian Refined Diagnosis Related Groups classification document (AR-DRGs) [42].

### Statistical analysis

Between group differences for primary and secondary outcome measures will be analysed using analysis of covariance (ANCOVA), suitable for use when extraneous factors need to be controlled.

Multivariate regression modelling will be used to examine predictors of successful response to intervention [43]. Potential predictors measured at baseline are personal characteristics, self efficacy beliefs, sub-domains of perceived health status, functional performance and disability levels whilst clinical improvements in NEADL and LLDI will be inputted as the dependant variable.

### Economic analysis

Incremental cost effectiveness analysis, that is the difference in mean cost to mean difference in effectiveness, will be the primary economic outcome [44]. Incremental cost per clinically significant improvement in the NEADL score will be determined, then incremental cost per QALY. Incremental QALY will be the sum of difference in duration of survival as weighted by quality of life between the two trial groups at three months. Confidence intervals will be calculated using Fieller’s method, and results will be presented in the form of a cost effectiveness acceptability curve [41]. A sensitivity analysis will be conducted to determine the ability to generalise results. The net costs and net effectiveness QALYS of HOME will be compared to hospital control group as expressed as incremental cost per QALY gained.

### Sample size calculation

A total of 400 participants will enter this two-armed, parallel-design study (200 per group). The probability is 80% that the study will detect a clinically important treatment difference at two-sided 0.05 significance level on either of the primary outcome measures. The NEADL is based on data from a trial by Logan et al. [45] on a between-group difference in the means of 1.73 and a standard deviation of 5.39 (NEADL score 0–22). Such a change would mean that clinically, an older person who was unable to walk around outside at the beginning of the study, would be able to do so at three months; or a person who may have needed help to do their own shopping at the beginning of the study would no longer require any assistance at three months. Adjustments have been made for drop-out (20%) and non-compliance (10%) based on pilot data [16]. Power analysis was conducted using Power and Precision Ver. 2.1.

## Discussion

The results of this study have the potential to change policy and occupational therapy practice within the acute healthcare system. A growing body of evidence supports the shift towards services that promote continuity of care across the hospital and community interface [8,30]. Currently, healthcare systems are known to be disjointed, have a lack of communication and inadequate co-ordination [46].

Our hypothesis is that participants of the HOME intervention will demonstrate higher levels of functional ability and will resume meaningful daily activities to a higher extent than the control group. Core to this is the belief that the determination of individual need should be contextually relevant and based on participant experience, and thus should include home assessment and that discharge planning should occur across the hospital to home divide [8]. The HOME protocol has been developed by chief investigators with a number of years of experience working with and developing intervention programs for older adults. The HOME protocol is based on current best practice and will now be evaluated for its effectiveness.

A unique contribution of this study is that the clinical effectiveness of a discharge planning program to reduce functional difficulties post-discharge will be established along with its cost and cost effectiveness. Including a cost effectiveness component in this study is important, as policy makers seek to maximise health care funds across populations [20]. Combining effectiveness of the HOME program with costs will enable policy makers and mangers to make informed decisions when allocating health care resources.

## Conclusion

This study will address a significant gap in current discharge planning and occupational therapy practice. The development of a best practice discharge planning program will be evaluated not only for its effectiveness but cost effectiveness as well.

## Competing interests

The authors declare that they have no competing interest.

## Authors’ contributions

KW collated the in-hospital and HOME protocols, and drafted the manuscript. LC jointly developed the in-hospital and HOME protocols, contributed to conceptualisation and study design, and contributed to the drafting of this manuscript. NL has contributed to the conceptualisation and study design, developed the in-hospital and HOME protocols, and contributed to the drafting of this manuscript. IC contributed to the conceptualisation of this study and design. GS has contributed to the cost effectiveness design. LG has contributed to the in-hospital and HOME protocols and study design. LR provided contributions to the conceptualisation and study design. SB contributed to the HOME protocol and study design. LM has contributed to the HOME protocol, CD contributed to the aims of the HOME protocol. All authors have reviewed and commented on this manuscript. All authors read and approved the final manuscript.

## Funding

This study is supported by a National Health and Medical Research Council Project Grant, 1009194.

## Pre-publication history

The pre-publication history for this paper can be accessed here:

http://www.biomedcentral.com/1471-2318/12/34/prepub
